# Hypo-Osmotic Swelling Test (HOST) for Feline Spermatozoa: The Simplified Procedure and the Aspect of Sperm Morphology

**DOI:** 10.3390/ani12070903

**Published:** 2022-03-31

**Authors:** Sylwia Prochowska, Wojciech Niżański, Alain Fontbonne

**Affiliations:** 1 Department of Reproduction and Clinic of Farm Animals, Wrocław University of Environmental and Life Sciences, pl. Grunwaldzki 49, 50-366 Wrocław, Poland; wojciech.nizanski@upwr.edu.pl; 2 École Nationale Vétérinaire d’Alfort, 7 Avenue du Général de Gaulle, 94700 Maisons-Alfort, France; alain.fontbonne@vet-alfort.fr

**Keywords:** cat, hypo-osmotic swelling test, water test, abnormal spermatozoa, clinical practice

## Abstract

**Simple Summary:**

One of the methods used to assess semen quality is the hypo-osmotic swelling test (HOST). Different protocols for this test are available, including a very simple one: swelling of spermatozoa is induced by incubation in distilled water for 5 min. Such a protocol could have easily been introduced in clinical practice, but it has not yet been evaluated for feline sperm cells. The objectives of this study were: (1) to check if a simplified HOST procedure can be applied for feline semen and (2) to check if sperm abnormalities may influence the results of this test. The results proved that the simplified HOST gives the same results as the standard one and that the presence of live, abnormal spermatozoa does not interfere with the test results.

**Abstract:**

Hypo-osmotic swelling test (HOST) is used to assess the functional integrity of sperm plasma membranes in many species. The primary aim of this study was to test a simplified HOST procedure for the evaluation of feline semen. The second objective was to check if sperm abnormalities can influence the results of this test. Urethral semen was collected from 19 male, domestic cats. In Exp. 1, HOST was performed in different media (50 mOsm/kg fructose or distilled water), temperature (37 °C or room temperature) and time (5 and 30 min). In Exp. 2, the potential effect of sperm abnormalities on HOST results was assessed by observing individual normal and abnormal spermatozoa microinjected into droplets of distilled water. The results showed no differences between the HOST results performed in different media, temperature and time. Viable abnormal spermatozoa were able to swell under hypo-osmotic conditions in the same manner as normal ones, except spermatozoa with distal droplets, which showed a higher frequency of ‘despiralization’. In conclusion, HOST can be reliably performed at 0 mOsm/kg for 5 min at room temperature, which may contribute to a wider use of this test under clinical environments. Viable abnormal spermatozoa are able to swell under hypo-osmotic conditions; therefore, their presence in the ejaculate would not bias the results of HOST when total coiling is calculated.

## 1. Introduction

The hypo-osmotic swelling test (HOS test, HOST) is widely used to assess the functional integrity of the sperm plasma membranes in both humans [1] and many animal species (reviewed by Zubair et al. [2]). The basis for this test is that only spermatozoa with an intact and functional cell membrane can maintain equilibrium between the intracellular and extracellular compartments and react to changes in the environment. Exposure to hypo-osmotic conditions causes an influx of fluid and swelling of the cell. Due to the special morphology of a spermatozoon, swelling is manifested not by an increase in cell size, but by coiling of the sperm tail. Therefore, coiled spermatozoa (HOST positive) are classified as membrane intact.

Despite its common use, there are some controversies regarding HOST. On the one hand, many studies proved its correlation with other sperm characteristics [3,4,5,6,7], as well as sperm fertilizing ability assessed in vitro and in vivo [4,5,8,9] and semen freezability [3,5]. On the other hand, some authors did not confirm these findings [10,11], which may be the result of species-specific differences and the lack of standardization of this test for particular species [12].

In fact, there are many different protocols available, varying in the osmolality of the medium used (ranging from 0 mOsm/kg to 150 mOsm/kg), the composition (fructose, lactose, sodium citrate, NaCl, TALP-HEPES) and the incubation time (from 2 to 60 min) [4,5,6,12,13,14]. Optimization studies have been performed in multiple species, including human [15], cat [6], dog [16], bull [17], horse [18], goat [19] and exotic species such as emu, Asian elephant, baboon, central rock rat [20] and honeybee [21]. These studies showed that for each species, maximum coiling is obtained under different conditions, especially with respect to osmolality (reviewed by Zubair et al. [2]). For example, for a domestic cat [6], the best results were obtained with 50 mOsm/kg fructose solutions and an incubation time of 30 min at 37 °C, whereas, for humans, a 150 mOsm/kg solution of fructose and sodium citrate mixture (50:50) and an incubation time of 5 or 30 min are recommended by the WHO [1].

Although HOST is very simple and inexpensive, the need for a special solution and incubator/water bath and the relatively long incubation time make this test impractical in clinical conditions. However, it has been shown that for human and animal spermatozoa (e.g., canine, bull, buck, honeybee), HOST can reliably be performed in distilled water for 5 min [13,14,21,22,23]; this is sometimes referred to as a ‘water test’ [21,22,23]. However, this protocol has not been tested in cats.

Spermatozoa subjected to hypo-osmotic conditions show different patterns of swelling, from minimal coiling on the tip of the tail, through various stages of bending and coiling, up to full coiling including the midpiece [15]. Some sperm defects (coiled tail, bent tail) already present in raw semen resemble these swelling patterns, and such abnormal sperm cells may be mistakenly classified as swollen [8]. On the other hand, it cannot be assumed that each abnormal spermatozoon possesses an impaired sperm membrane—to the authors’ knowledge, there is no study examining whether viable abnormal spermatozoa react to hypo-osmotic conditions in the same manner as normal ones. Domestic cats are well known for the high percentage of morphologically abnormal spermatozoa in their semen, with mean tail defects ranging up to 20% [6,24], and therefore, investigating this aspect may be especially important in this species.

The primary aim of this study was to simplify the HOST procedure for the evaluation of feline semen by testing distilled water as a hypo-osmotic solution together with different temperatures and time of incubation. The secondary aim was to check if the presence of sperm abnormalities influences the results of this test.

## 2. Materials and Methods

### 2.1. Semen Collection

Urethral semen was collected from 19 male, domestic cats (different breeds, aged 9 months to 6 years) according to Zambelli et al. [25] with additional transscrotal stimulation [26]. Briefly, medetomidine hydrochloride (Sedator, 1.0 mg/mL, Novartis, Poland) was administered intramuscularly at a dose of 100 µg/kg. Five to ten minutes after the sedation effect was reached, a transscrotal massage of caudae epididymides was applied for 2 min. Then, a semen sample was collected by placing an open-end tomcat urethral catheter 8–9 cm into the urethra. After a few seconds, the catheter was withdrawn and the collected spermatozoa were placed in an Eppendorf tube containing 200 µL of pre-warmed TRIS-based semen extender (3.02% (*w*/*v*) TRIS, 1.35% (*w*/*v*) citric acid, and 1.25% (*w*/*v*) fructose in bidistilled water; pH 6.5). All laboratory chemicals and reagents used in this study were purchased from Sigma-Aldrich, Poznań, Poland, unless stated otherwise. Semen collection procedures were approved by the Local Ethical Committee in Wroclaw, Resolution no. 044/2020.

### 2.2. Semen Assessment

Sperm samples were assessed for subjective motility with the use of a phase contrast microscope. Then, total sperm count and motility parameters were evaluated with the use of a computer-assisted semen analyzer (CASA-system, HTM-IVOS, 12.3D, Hamilton-Thorne Biosciences, Beverly, MA, USA). The procedure and software settings have been described previously [24].

Viability and morphology were assessed on eosin-nigrosine-stained slides as previously described [24]. Viability was estimated by counting 200 spermatozoa, and then another 200 spermatozoa were assessed morphologically. Spermatozoa with red coloration on the head were considered dead and sperm cells were considered abnormal if they possessed at least one of the following: abnormal head size or shape, detached head, midpiece defects, bent or coiled tail or proximal or distal droplet.

### 2.3. Exp. 1—Hypo-Osmotic Swelling Test (HOST) under Different Conditions

HOST was performed by adding 5 μL of semen to 50 μL of hypo-osmotic solution (50 mOsm/kg fructose or 0 mOsm/kg distilled water) and incubating samples at 37 °C or room temperature for 5 or 30 min. The combination of these conditions created six experimental groups:

(1) 50 mOsm/kg fructose, 37 °C, 5 min
(2) 50 mOsm/kg fructose, 37 °C, 30 min
(3) Distilled water (0 mOsm/kg), 37 °C, 5 min
(4) Distilled water (0 mOsm/kg), 37 °C, 30 min
(5) Distilled water (0 mOsm/kg), room temperature, 5 min
(6) Distilled water (0 mOsm/kg), room temperature, 30 min

**Figure 1:**
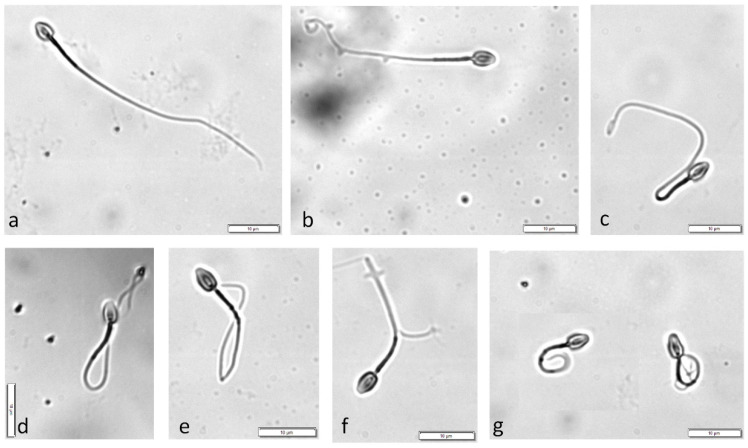
Different morphological changes observed in cat spermatozoa exposed to hypo-osmotic solutions—representative pictures. Coiling patterns classification according to WHO manual [1]. (**a**) spermatozoon HOST negative (tail not coiled). (**b**–**g**) spermatozoa classified as HOST positive (increasing coiling of the tail from **b** to **g**). (Phase contrast microscope, ×1000. White bars represent 10 µm.)

After incubation, a 10 μL sample from each experimental group was placed on a microscopic slide with a cover slip and 200 spermatozoa were classified as HOST positive (coiling pattern b–g, Figure 1b–g) or HOST negative (not coiled, Figure 1a). Semen from 13 cats was used.

### 2.4. Exp. 2—Microinjection of a Single Spermatozoon into a Droplet of Distilled Water

To analyze the effect of sperm defects on swelling capacity in hypo-osmotic conditions, the microinjection of a single spermatozoon into a droplet of distilled water was performed. Briefly, 2 μL of semen was transferred to a 10 µL drop of HTF, linked to the 5 µL ridge of a SpermCatch^®®^ (Irvine Scientific, Newtownmountkennedy, Co. Wicklow, Ireland) under mineral oil on a 5 cm Petri dish, and incubated for 15 min at 37 °C and 5% CO₂. Then, a chosen motile (presumably membrane intact) spermatozoon was aspirated into the microinjection pipette (with an inner diameter of 5 µm, Microtech IVF, s.r.o. Brno, Czech Republic), transferred to a 10 µL drop of distilled water on the same Petri dish and observed for approximately 1 min. For each spermatozoon, the type of morphological abnormality (bent tail, coiled tail, abnormal midpiece, proximal droplet, distal droplet or no abnormality—normal) was noted, along with the type of reaction. Semen from 6 cats was used, and at least 50 spermatozoa from each cat were evaluated. We tried to balance the morphology groups numerically; however, due to the differing percentage of sperm abnormalities in each cat, sometimes this was not possible (e.g., no distal and proximal droplets in one cat, 30% of bent tails in the other). In addition, some midpiece defects, as well as Dag-like defects, made it impossible to aspirate the spermatozoon into the pipette; therefore, not all sperm defects could be evaluated. In total, 339 spermatozoa were assessed, with the distribution of spermatozoa as follows: 67 normal, 111 bent tail, 45 coiled tail, 48 abnormal midpiece, 41 proximal droplets and 27 distal droplets.

### 2.5. Statistical Analysis

Statistical analysis was performed using R Statistical Software (Foundation for Statistical Computing, Vienna, Austria). The data distribution was evaluated using the Shapiro–Wilk test. As the data were not distributed normally, Friedman ANOVA was performed to detect the combined effect of osmolarity, temperature and time. Spearman rank correlation was performed to analyze the correlation between membrane integrity assessed by HOST and subjective motility, morphology and viability assessed by eosin-nigrosine staining. The effect of sperm morphology in Exp. 2 was determined by Fisher’s exact test with Monte Carlo estimation. Post hoc analysis was performed with multiple pairwise tests, with Benjamini–Hochberg correction of the *p*-value. The level of significance was set at *p* < 0.05, except for post hoc analyses in Exp. 2, where it was set at *p* < 0.1.

## 3. Results

### 3.1. Exp. 1—Effect of Medium, Temperature, and Time

The quality of semen used in this experiment was as follows (mean ± SD): total sperm count 25.4 ± 16.3 × 10^6^, subjective motility 58.0 ± 23.7%, viability 85.0 ± 11.9%, normal morphology 34.2 ± 19.0%. Detailed motility parameters obtained by CASA are shown in Table 1.

**Figure 2:**
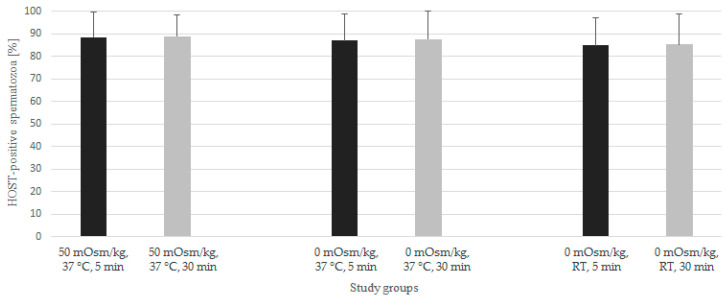
The results of Exp. I—hypo-osmotic swelling test (HOST) performed in different media (50 mOsm/kg fructose or 0 mOsm/kg distilled water) incubated at different temperatures (37 °C or room temperature—RT) for 5 min or 30 min. Results presented as mean ± SD (*n* = 13). No statistically significant differences were noted.

The results of HOST performed under different conditions are presented in Figure 2. No statistically significant differences were found between the variables evaluated. The results of HOST in each combination were significantly correlated with the viability assessed by eosin-nigrosine (r > 0.75, *p* < 0.05), but not with subjective motility and morphology (*p* > 0.05).

### 3.2. Exp. 2—Effect of Sperm Morphology

The quality of semen used in this experiment was as follows (mean ± SD): total sperm count 22.5 ± 23.6 × 10^6^, subjective motility 54.0 ± 30.7%, viability 78.5 ± 12.0%, normal morphology 42.2 ± 15.9%. The most common morphological abnormality was a bent tail (15.4 ± 10.9%), followed by an abnormal midpiece (10.3 ± 5.3). Detailed motility parameters obtained by CASA are shown in Table 1.

During this experiment, four types of reaction to hypo-osmotic conditions were observed:

- Immediate full coiling (Video S1)—coiling of sperm tail up to the head within 10 s;
- Despiralization (named after Drevius and Eriksson 1966 [27]) (Video S2)—immediate coiling and then rapid, partial uncoiling within 15 s;
- Atypical coiling—either not full or not immediate (Video S3);
- No reaction (Video S4).

**Figure 3:**
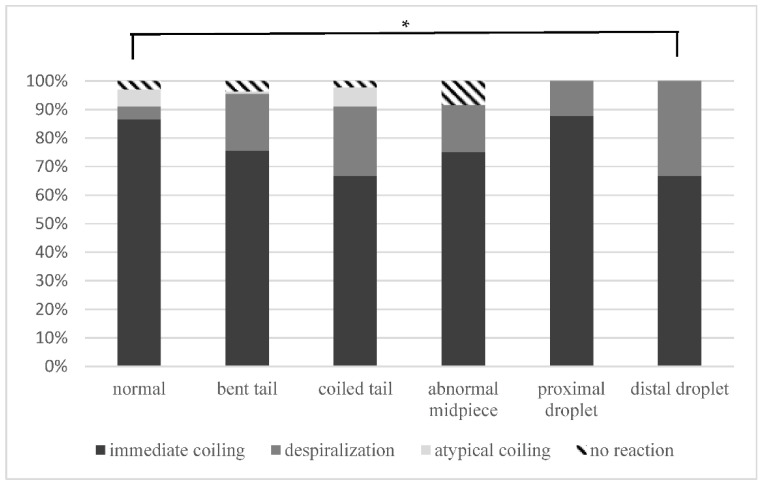
The results of Exp. II—the percentage of different reaction types of single motile spermatozoa placed in distilled water in each morphology group. Asterisk (*) indicates a significant difference at *p* < 0.1.

The proportion of different reactions in each morphology group is presented in Figure 3. No statistically significant differences were found, except when comparing the despiralization pattern in spermatozoa with distal droplets to normal ones. Immediate full coiling was the most common pattern (>60%), regardless of sperm morphology. Abnormal spermatozoa were able to swell under hypo-osmotic conditions, and after coiling, they were indistinguishable from coiled normal spermatozoa. Spermatozoa that uncoiled after a few seconds (despiralized) remained bent and never reached full straightening. Some spermatozoa did not swell (no reaction); however, they became immotile in the micropipette, and it is possible that they were damaged during aspiration.

## 4. Discussion

This study confirmed the possibility of using a simplified HOST procedure (performed in distilled water for a short time at room temperature) for the evaluation of membrane functionality of feline spermatozoa. Another important observation was that viable abnormal spermatozoa swell in hypo-osmotic conditions comparably to normal ones.

Most of the HOST protocols include an incubation time of 30–60 min, which is based on the observation that beyond this time, the percentage of coiled sperm cells does not change [28]. Conversely, water tests are commonly performed within 5 min, because there is a fear that prolonged exposure to such an unfavorable environment will result in cell death [22]. The WHO recommends 5 min of incubation time when spermatozoa are to be processed for therapeutic use (e.g., selecting viable spermatozoa for ICSI in patients with asthenozoospermia) and 30 min of incubation for routine diagnostics [1]. The results of this study showed no differences between 5 and 30 min of incubation time, regardless of the medium used, and therefore, shortened protocols for both classic HOST and the water test can be used in domestic cats. Additionally, results showed that prolonged exposure to distilled water did not cause further cell death; therefore, the incubation time can be extended if needed.

In human medicine, when evaluating HOST results, different types of coiling are noted separately [23,28,29,30]; however, most animal studies [6,7,9,10,13,20] and the WHO manual [1] take into consideration the total percentage of positive cells, regardless of the type of coiling. This reflects how it was performed in this study in Exp. 1. When exposed to hypo-osmotic conditions, the spermatozoa showed different swelling patterns named ‘a’ to ‘g‘, where ‘a’ represents no reaction (HOST negative), ‘b’ depicts swelling only at the tail-tip, ‘g’ represents full swelling up to the head of the sperm, and ‘c’–‘f’ are stages in between these opposing patterns [15]. In Exp. 1, all types were observed, whereas in Exp. 2, the predominant reaction was immediate full coiling (‘g’ type). This is not surprising, as it is well known from very early studies [27] that lowering the medium tonicity will result in stronger tail coiling.

When the osmotic resistance of the cell is exceeded, it causes ‘despiralization’—a sudden, partial straightening of the sperm tail—which can be interpreted as rupture of the cell membrane. This is contrary to the slow uncoiling observed in sperm cells returned to isotonicity [27]. Despiralization was observed in Exp. 2 as well. It is interesting that Drevius and Eriksson [27] reported that only a low percentage of bull sperm cells remain coiled in 0.015 M NaCl (osmolarity 30 mOsm/kg) and further addition of water caused their immediate despiralization. In our study, most of the feline spermatozoa immersed in water presented full coiling and only 4.5% (normal spermatozoa) to 33.3% (distal droplets) of sperm cells despiralized. This could be partially due to time (Drevius and Eriksson observed despiralization after around 1.5–2 min [27]); however, studies in humans [23] and dogs [14] reported a high percentage of fully coiled spermatozoa species in water test reads after 5 min of incubation. This may suggest that bull spermatozoa have lower resistance to hypo-osmotic conditions than feline or canine spermatozoa, but further studies are required to confirm this hypothesis. What needs to be highlighted is that despiralized spermatozoa remain partially coiled/bent; therefore, as long as total coiling is assessed, this phenomenon does not affect the results of the water test.

Scoring the type of coiling, instead of assessing the total coiling, may be beneficial in some cases. In humans, type ‘g’ showed the highest chance of having abnormal sperm head and damaged acrosome [29], damaged DNA and apoptotic changes [30], and it is suggested not to use this type of spermatozoa for ICSI [30]. It must be highlighted that the ratio of particular coiling types changes with decreasing osmolality [14,23,27] and incubation time [28], showing that a standardized procedure must be applied when coiling types are assessed. Therefore, the simplified HOST procedure investigated in this study can be used only with total coiling calculation. However, we studied a simplified protocol for use in general veterinary practice. In this case, total swelling is sufficient for basic semen analysis. Additionally, as the ‘g’ type is the most evident sign of swelling, the use of distilled water, which causes stronger coiling, may facilitate assessment by less experienced persons such as general vet practitioners.

HOST is performed mainly at 37–38.5 °C, probably due to common findings that sperm cells are very susceptible to cold shock. Therefore, good laboratory practice is to keep semen at body temperature. However, it has been shown for buck semen that a handling temperature of 20 °C does not negatively influence spermatozoa [31], and the WHO manual [1] specifies that the semen specimen can be placed on the bench (room temperature) for liquefaction (lasting 30–60 min). There may be species-specific differences in cold-shock resistance, but, to the authors’ knowledge, an optimal handling temperature for feline spermatozoa has not yet been established. The results of this study showed that temperature did not influence the results of the water test, proving that it can easily be performed at room temperature.

The topic regarding how inherent defects in the sperm tail may affect the results of HOST has generally been neglected in studies. In some studies, individuals with a high percentage of sperm defects, mainly coiled tails, were rejected from the study [15,19], which cannot be done in the case of samples from patients. In others [4,8], the percentage of tail defects assessed in the original sample was subtracted from the percentage of swollen sperm cells in HOST. Our study showed that motile (presumptively live, membrane-intact) abnormal sperm cells react ‘normally’ to the hypo-osmotic conditions; therefore, such an approach (extraction of the entire abnormal pool from the total coiled group) seems to be inappropriate. However, non-functional abnormal spermatozoa may still bias the results. The solution for this may be simultaneous HOST and eosin Y staining [32] or eosin-nigrosine staining [13,33]. Live spermatozoa actively exclude eosin dye and therefore remain unstained, contrary to dead ones, whose heads appear reddish/pink. Although damage to the head and tail may occur separately, this is a rare situation [33]. Therefore, when a coiled spermatozoon possesses a stained head, it can be stipulated that coiling is an inherited abnormality, not due to the effect of swelling. Another solution may be the immersion of spermatozoa in a hypo-osmotic medium with a high refractive index (e.g., Ficoll solution) and its evaluation under a positive phase contrast [27]. In such a test, the swelling area is easily visible as a bright vesicle surrounding coiled parts [27]. Both modifications negate the idea of protocol simplification for use in clinical conditions, but these can be introduced in the andrological laboratory.

## 5. Conclusions

For feline spermatozoa, HOST can be reliably performed in 0 mOsmol/kg for 5 min at room temperature. Shortened time, incubation at ambient temperature, and the use of easily accessible, distilled water may lead to the wide use of this test in clinical conditions. Viable abnormal spermatozoa swell in hypo-osmotic conditions comparably to normal ones, showing that they maintain their membrane functional integrity.

## Figures and Tables

**Table 1:** Sperm motility parameters obtained by CASA of semen used in Exp. 1 and Exp. 2. Results presented as mean ± SD.

CASA Parameter	Exp. 1 (*n =* 13)	Exp. 2 (*n* = 5)
VAP (µm/s)	100.8 ± 41.7	111.3 ± 45.7
VSL (µm/s)	80.7 ± 43.4	95.3 ± 48.8
VCL (µm/s)	158.7 ± 50.4	163.4 ± 81.6
ALH (µm)	7.0 ± 1.2	6.1 ± 2.8
BCF (Hz)	25.9 ± 5.1	26.7 ± 11.4
STR (%)	73.8 ± 14.6	70.4 ± 33.0
LIN (%)	47.5 ± 14.2	48.3 ± 20.9
ELONGATION (%)	78.1 ± 10.5	68.3 ± 32.3
MOT (%)	58.6 ± 23.8	53.4 ± 31.2
PMOT (%)	26.9 ± 19.5	34.1 ± 23.2
RAPID (%)	41.4 ± 17.9	41.6 ± 27.6
SLOW (%)	35.4 ± 19.2	33.4 ± 22.4
STATIC (%)	2.1 ± 2.0	3.7 ± 6.2

VAP—average path velocity; VSL—straight line velocity; VCL—curvilinear velocity; ALH—amplitude of lateral head displacement; BCF—beat cross frequency; STR—the straightness of movement; LIN—the linearity of movement. MOT—the percentage of motile spermatozoa; PMOT—the percentage of spermatozoa with a progressive motility; RAPID—subpopulations of spermatozoa showing rapid movement; SLOW—subpopulations of spermatozoa showing slow movement; STATIC—subpopulations of static spermatozoa.

## Data Availability

Data supporting the findings of this study are available from the corresponding author (S.P.), upon reasonable request.

## Supplementary Materials

The following supporting information can be downloaded at: https://www.mdpi.com/article/10.3390/ani12070903/s1, Video S1: Hypo-osmotic coiling pattern—immediate full coiling. Video S2: Hypo-osmotic coiling pattern—despiralization. Video S3: Hypo-osmotic coiling pattern—atypical coiling. Video S4: Hypo-osmotic coiling pattern—no reaction.
